# Priming locomotor training with transspinal stimulation in people with spinal cord injury: study protocol of a randomized clinical trial

**DOI:** 10.1186/s13063-023-07193-4

**Published:** 2023-02-25

**Authors:** Andreas Skiadopoulos, Grace O. Famodimu, Shammah K. Solomon, Parul Agarwal, Noam Y. Harel, Maria Knikou

**Affiliations:** 1 Klab4Recovery Research Program, The City University of New York, College of Staten Island, Staten Island, NY USA; 2 Department of Physical Therapy, College of Staten Island, The City University of New York, Staten Island, NY USA; 3 Spinal Cord Damage Research Center, James J. Peters Department of Veterans Affairs Medical Center, Bronx, NY USA; 4 Population Health Science & Policy, Institute for Health Care Delivery Science, Icahn School of Medicine at Mount Sinai, Manhattan, NY USA; 5 PhD Program in Biology and Collaborative Neuroscience Program, Graduate Center of The City University of New York and College of Staten Island, Manhattan & Staten Island, NY USA

**Keywords:** Transspinal stimulation, Locomotor training, Spinal cord injury, Neurophysiology, Standing, Stepping, Rehabilitation, Combined interventions

## Abstract

**Background:**

The seemingly simple tasks of standing and walking require continuous integration of complex spinal reflex circuits between descending motor commands and ascending sensory inputs. Spinal cord injury greatly impairs standing and walking ability, but both improve with locomotor training. However, even after multiple locomotor training sessions, abnormal muscle activity and coordination persist. Thus, locomotor training alone cannot fully optimize the neuronal plasticity required to strengthen the synapses connecting the brain, spinal cord, and local circuits and potentiate neuronal activity based on need. Transcutaneous spinal cord (transspinal) stimulation alters motoneuron excitability over multiple segments by bringing motoneurons closer to threshold, a prerequisite for effectively promoting spinal locomotor network neuromodulation and strengthening neural connectivity of the injured human spinal cord. Importantly, whether concurrent treatment with transspinal stimulation and locomotor training maximizes motor recovery after spinal cord injury is unknown.

**Methods:**

Forty-five individuals with chronic spinal cord injury are receiving 40 sessions of robotic gait training primed with 30 Hz transspinal stimulation at the Thoracic 10 vertebral level. Participants are randomized to receive 30 min of active or sham transspinal stimulation during standing or active transspinal stimulation while supine followed by 30 min of robotic gait training. Over the course of locomotor training, the body weight support, treadmill speed, and leg guidance force are adjusted as needed for each participant based on absence of knee buckling during the stance phase and toe dragging during the swing phase. At baseline and after completion of all therapeutic sessions, neurophysiological recordings registering corticospinal and spinal neural excitability changes along with clinical assessment measures of standing and walking, and autonomic function via questionnaires regarding bowel, bladder, and sexual function are taken.

**Discussion:**

The results of this mechanistic randomized clinical trial will demonstrate that tonic transspinal stimulation strengthens corticomotoneuronal connectivity and dynamic neuromodulation through posture-dependent corticospinal and spinal neuroplasticity. We anticipate that this mechanistic clinical trial will greatly impact clinical practice because, in real-world clinical settings, noninvasive transspinal stimulation can be more easily and widely implemented than invasive epidural stimulation. Additionally, by applying multiple interventions to accelerate motor recovery, we are employing a treatment regimen that reflects a true clinical approach.

**Trial registration:**

ClinicalTrials.gov NCT04807764. Registered on March 19, 2021.

**Supplementary Information:**

The online version contains supplementary material available at 10.1186/s13063-023-07193-4.

## Administrative information

Note: the numbers in curly brackets in this protocol refer to SPIRIT checklist item numbers. The order of the items has been modified to group similar items (see http://www.equator-network.org/reporting-guidelines/spirit-2013-statement-defining-standard-protocol-items-for-clinical-trials/).

**Table:** 

Title {1}	Priming locomotor training with transspinal stimulation in people with spinal cord injury: protocol of a randomized clinical trial
Trial registration {2a and 2b}.	ClinicalTrials.gov: NCT04807764
Protocol version {3}	- Issue Date: May 31, 2022 - Protocol amendment number: 2 - Authors: Maria Knikou and Noam Y. Harel
Funding {4}	Study is sponsored by the Eunice Kennedy Shriver National Institute of Child Health and Human Development (NICHD) / National Institutes of Health (NIH); Award No: R01HD100544
Author details {5a}	Andreas Skiadopoulos ^1,2^, Grace O. Famodimu ^3^, Shammah K. Solomon ^1,2^, Parul Agarwal ^4^, Noam Y. Harel ^3,4^, Maria Knikou ^1,2,5^ ^1^ Klab4Recovery Research Program, The City University of New York, College of Staten Island, New York, USA ^2^ Department of Physical Therapy, College of Staten Island, The City University of New York, Staten Island, New York, USA ^3^ Spinal Cord Damage Research Center, James J. Peters Department of Veterans Affairs Medical Center, Bronx, New York, USA ^4^ Population Health Science & Policy, Institute for Health Care Delivery Science, Icahn School of Medicine at Mount Sinai, Manhattan, New York, USA ^5^ PhD Program in Biology and Collaborative Neuroscience Program, Graduate Center of The City University of New York and College of Staten Island, New York, USA
Name and contact information for the trial sponsor {5b}	Joe Bonner, Ph.D. Health Scientist Administrator / Program Officer National Center for Medical Rehabilitation Research (NCMRR) National Institute of Child Health and Human Development (NICHD) BG 6710 Rockledge DR Wing B RM 2114 6710B Rockledge DriveE Bethesda MD 20,817 E-mail: joe.bonner@nih.gov Phone: (301) 827–8303
Role of sponsor {5c}	The sponsor did not contribute to the study design, data collection, management, analysis, and interpretation. The sponsor did not contribute to the decision to submit this report for publication and does not have ultimate authority over any of the authors’ activities.

## Introduction

### Background and rationale {6a}

Spinal cord injury (SCI) is a long-term disability that greatly impacts quality of life for nearly 27 million people globally, and about 300,000 individuals in the USA [1, 2]. Restoration of motor function and especially stepping ability is of high priority for individuals with SCI wanting to improve functional independence and enhance quality of life [3]. In addition to the clinical sequelae of paresis and ambulatory dysfunction, SCI manifests as defects in multiple neurophysiological biomarkers, including but not limited to cortically induced motor evoked potentials (MEPs) and spinally induced Hoffmann (H) reflexes [4, 5]. MEPs provide information on the integrity of synaptic connections between the brain and spinal cord, while H reflexes provide information regarding spinal reflex excitability and function of complex spinal neuronal networks necessary for human movement. After SCI, MEPs have significantly delayed onsets, decreased amplitudes, and increased thresholds [4, 6]. SCI also hinders the ability of descending volleys to summate at the spinal cord and limits the electrophysiological transmission of uninjured axons [7, 8]. Damage to spinothalamic and dorsal column circuits disrupts transmission from receptors registering muscle stretch and cutaneous sensation [9]. These disruptions in the function and neurotransmission of uninjured fibers clearly demonstrate that SCI induces pathophysiology in the central nervous system beyond the immediate area of damage [10–14]. Consequently, interventions that effectively promote appropriate neuromodulation of spinal locomotor networks and strengthen connectivity of distributed neuronal networks across the neural axis are greatly needed.

Non-invasive rehabilitation strategies that restore or improve the ability of stepping have gained substantial interest in the research and clinical communities [15–17]. One of the most prevalent rehabilitation strategies is body weight support (BWS) step training with either manual or robotic assistance of leg movements. This strategy utilizes movement-induced afferent feedback to promote activity-dependent neuronal reorganization of neuronal networks after SCI. Afferent feedback, and the spinal reflex circuits integrating afferent feedback, is an essential source of the locomotor control scheme that alters the action of spinal locomotor networks to refine and coordinate the activity of different muscles across joints and between limbs during stepping [18, 19]. This is evident with the phase-dependent modulation of muscle and cutaneous reflexes that facilitate stepping such as the soleus H- and stretch-reflexes and the tibialis anterior (TA) flexion reflex [10, 20–23]. The important role of afferent feedback to spinal locomotor network function is further supported by the phase-dependent modulation of primary afferent depolarization and presynaptic inhibition of afferent feedback [24, 25]. SCI disrupts the phase-dependent modulation of the soleus H-reflex during walking [10], while the most common change observed with BWS-assisted step training is the partial return of soleus H-reflex depression during the swing phase [26]. Additional changes include restoration of soleus H-reflex rate-dependent or homosynaptic depression, recovery of spinal inhibitory circuits and spinal reflex excitability, and a more physiological intralimb and interlimb muscle coordination [27, 28]. These changes are likely linked to the normalization of the proportion of excitatory and inhibitory synaptic inputs to spinal motoneurons [29, 30], improvements in synaptic inputs from Ia afferents [31], and alterations in the concentration of neurotransmitters [32].

Transcutaneous spinal cord (transspinal) stimulation over the thoracolumbar region is a non-invasive, cost-effective method to produce neuromodulation across broad spinal neural networks. Transspinal stimulation facilitates recovery of motor, including locomotor, function after SCI [33, 34], increases the excitability of the spinal networks deprived of descending input, and enhances their responsiveness to input from residual intact descending pathways and afferent feedback [35]. Of clinical importance, transspinal stimulation enables the activation of spinal locomotor networks, aids in the generation of step-like muscle activity [36, 37], increases the net motor output [33], and reduces hyperreflexia [38–40] in individuals with SCI. The specific neuromodulation mechanisms likely incorporate the excitation of posterior root afferents which transsynaptically activate spinal locomotor networks, including spinal interneurons and motoneurons. Interestingly, transspinal evoked potentials (TEPs) and proprioceptive reflexes share similar neurophysiological properties [41–43]. For example, soleus H-reflexes and soleus TEPs are susceptible to rate-dependent homosynaptic depression when single stimuli at low frequencies are used, and when pairs of transspinal stimuli are delivered at short interstimulus intervals [42, 44]. Furthermore, TEPs recorded from the soleus muscle are depressed by Achilles tendon vibration and activation of the antagonist tibialis anterior (TA) muscle [45], and are modulated in a similar phase-dependent pattern in individuals with and without SCI during stepping [43, 46]. Together, these findings suggest that TEPs are conveyed by similar neuronal pathways to those used by the soleus H-reflex.

Locomotor training has been coupled with transcranial and/or transspinal direct current stimulation [47–49]. Studies have assessed the simultaneous combination of transspinal stimulation during locomotor training [40, 50–52]. In other contexts, such as upper extremity training after stroke, stimulation to the cortex applied prior to exercise may act as a “primer” to increase the neuroplastic response to exercise [53, 54]. The synergistic use of transspinal stimulation to prime locomotor training is a novel rehabilitation approach with significant clinical importance. In this randomized clinical trial, we test the effects of priming locomotor training with transspinal stimulation to maximize improvements in standing and walking ability in response to 40 sessions of therapy.

### Objectives {7}

The overall objective of this mechanistic randomized clinical trial is to use transspinal stimulation over the thoracolumbar enlargement, the spinal location of leg motor circuits, to prime locomotor training and ultimately improve the ability to stand and walk in individuals with chronic incomplete SCI. We hypothesize that non-invasive thoracolumbar transspinal stimulation administered before locomotor training increases neuronal responsiveness over multiple spinal segments, mechanistically reflected by increased responses to motor cortex stimulation, and improved phase-dependent modulation of spinal reflexes during walking. Because transspinal stimulation facilitates anti-gravity neuronal circuits, we further hypothesize that transspinal stimulation delivered while participants stand will augment gains compared to delivery in the supine position. To meet our overall objective, 45 individuals with incomplete SCI will undergo 40 sessions of BWS step training primed with 30 Hz transspinal stimulation. Participants are randomized to receive transspinal stimulation during standing (active or sham) or while supine (active).

Our *specific objective 1* determines whether priming locomotor training with transspinal stimulation strengthens corticomotoneuronal connectivity through posture-dependent corticospinal neuroplasticity, as reflected by increased MEP amplitudes and phase-dependent MEP amplitude modulation during stepping. We hypothesize that active but not sham transspinal stimulation delivered during standing increases MEP amplitudes and improves phase-dependent MEP amplitude modulation during stepping. We further hypothesize that transspinal stimulation delivered while supine increases MEP amplitudes but does not induce phase-dependent MEP amplitude modulation during stepping.

Our *specific objective 2* determines if priming locomotor training with transspinal stimulation evokes appropriate neuromodulation of spinal locomotor networks, as reflected by restored spinal inhibition, reduced neurophysiological hyperreflexia, and re-established phase-dependent soleus H-reflex modulation during stepping. We hypothesize that active but not sham transspinal stimulation delivered during standing (1) restores soleus H-reflex rate-dependent depression, (2) increases the depth of reciprocal inhibition between ankle extensor and flexor muscles, and (3) improves soleus H-reflex phase-dependent modulation towards a more physiological pattern during stepping. We further hypothesize that rate-dependent soleus H-reflex depression and soleus H-reflex modulation during assisted stepping recovers more in the transspinal-standing group than in the transspinal-supine group.

Our *specific objective 3* determines if priming locomotor training with transspinal stimulation improves the ability to stand and walk. Comparing locomotor electromyography (EMG) activity patterns before and after therapy will measure reorganized motoneuron activity and return of reciprocal activation between antagonistic muscles supporting intralimb and interlimb coordination. We hypothesize that the number of motor modules increases in the transspinal-standing group, and that antagonistic muscles show more reciprocal inhibition during stepping.

Our *specific objective 4* establishes clinically functional gains in motor and autonomic function. Improvements in walking ability are assessed with the 10-m test, 2-min test; improvements in balance ability are assessed with the BESTtest. Improvements in bowel, bladder, and sexual function are assessed with questionnaires. Clinical outcomes will be correlated with neurophysiological outcomes to identify predictors of recovery that can be based both on electrophysiological biomarkers and clinical assessment outcomes.

Upon conclusion, this clinical trial will indicate that priming locomotor training with transspinal stimulation improves the ability to stand and walk by facilitation and better synchronization of spinal locomotor neuronal networks and reflex circuits subserving standing and walking. This finding will support that transspinal stimulation works in parallel and not antagonistic to exercise, and can constitute a new standard of care for people with chronic SCI.

### Trial design {8}

This is a mechanistic, sham-controlled, randomized, parallel design, multi-site (2 sites), clinical trial in which participants with chronic SCI are assigned in equal ratios to one of three study groups (or arms). Group 1: Active transspinal stimulation while standing followed by locomotor training; Group 2: Active transspinal stimulation while lying supine followed by locomotor training; and Group 3: Sham transspinal stimulation while standing followed by locomotor training. Study outcomes are assessed for superiority before (baseline) and after 40 sessions of primed locomotor training.

## Methods: participants, interventions, and outcomes

### Study setting {9}

Neurophysiological assessments before and after 40 sessions of therapy are performed at the KLab4Recovery Research Program located at the College of Staten Island, City University of New York, New York, USA, an academic institution. Therapy and clinical assessments are performed at the KLab4Recovery Research Program and at one clinical research center (Spinal Cord Damage Research Center) of a government hospital (James J. Peters Veterans Affairs Medical Center, Bronx, New York USA). Both study sites are listed at ClinicalTrials.gov (https://clinicaltrials.gov/ct2/show/NCT04807764).

### Eligibility criteria {10}

The inclusion criteria for the participants are the following:

◦ Willingness to comply with all study procedures and availability for the duration of the study.
◦ Ability to understand and sign informed consent.
◦ Male or female, age 18–70 years old.
◦ A diagnosis of first time SCI due to trauma, vascular or orthopedic pathology.
◦ At least 6 months after SCI.
◦ American Spinal Injury Association Impairment Scale (AIS) Grade C or D.
◦ Minimum bone mineral density − 2.49.
◦ Lesion at or above Thoracic 10 neurological level.
◦ Absent lower motor neuron injury.
◦ Presence of tendon reflexes.
◦ Stable medical condition without cardiopulmonary disease or cognitive impairment.
◦ Absent permanent ankle joint contractions that prevent passive or active ankle movement.

The exclusion criteria for the participants are the following:

◦ Supraspinal lesions or disease.
◦ Neuropathies of the peripheral nervous system.
◦ Degenerative neurological disorders of the spine or spinal cord.
◦ Neoplastic or vascular disorders of the spine or spinal cord.
◦ Urinary tract infection.
◦ Presence of pressure sores.
◦ Permanent ankle joint contractures that prevent passive or active ankle movement.
◦ Participation in another research study or new rehabilitation program.
◦ Pregnant women or women who suspect they may be pregnant or may become pregnant.
◦ People with cochlear implants, pacemaker, implanted infusion device, and/or implanted stimulators of any type.
◦ People with a history of seizures or medical conditions or medications that increase the possibility of seizures.

### Who will take informed consent? {26a}

Study personnel approved by the Institutional Review Board (IRB) at each study site obtain informed consent according to the principles of the Declaration of Helsinki. All research personnel are trained and certified in policies of privacy, confidentiality, and data integrity.

### Additional consent provisions for collection and use of participant data and biological specimens {26b}

Not applicable. No biological specimens are collected for research purposes. Therefore, no additional consent is obtained from the participants.

## Interventions

### Explanation for the choice of comparators {6b}

There are two active and one sham stimulation interventions delivered to three parallel groups. Active transspinal stimulation is delivered while standing with BWS and/or in the supine position. Sham transspinal stimulation is delivered while standing with BWS. All participants undergo the same locomotor training intervention. The guarantee of locomotor training and the 2:1 ratio of active to sham stimulation reduce the risk of people refusing to participate in the trial. For the sham protocol, the stimulator frequency and waveform is identical to active stimulation, but the intensity is held at the motor threshold for only 1 min, followed by a gradual return to no stimulation for the subsequent 29 min. Studies have suggested that this pattern of stimulation can mimic the initial sensation of stimulation followed by the acclimation to cutaneous stimulation. This approach is expected to improve blinding to active versus sham stimulation.

### Intervention description {11a}

The intervention is 30 min of 30 Hz thoracolumbar transspinal stimulation followed by 30 min of robotic gait training.

#### Transspinal stimulation

**Fig. 1:**
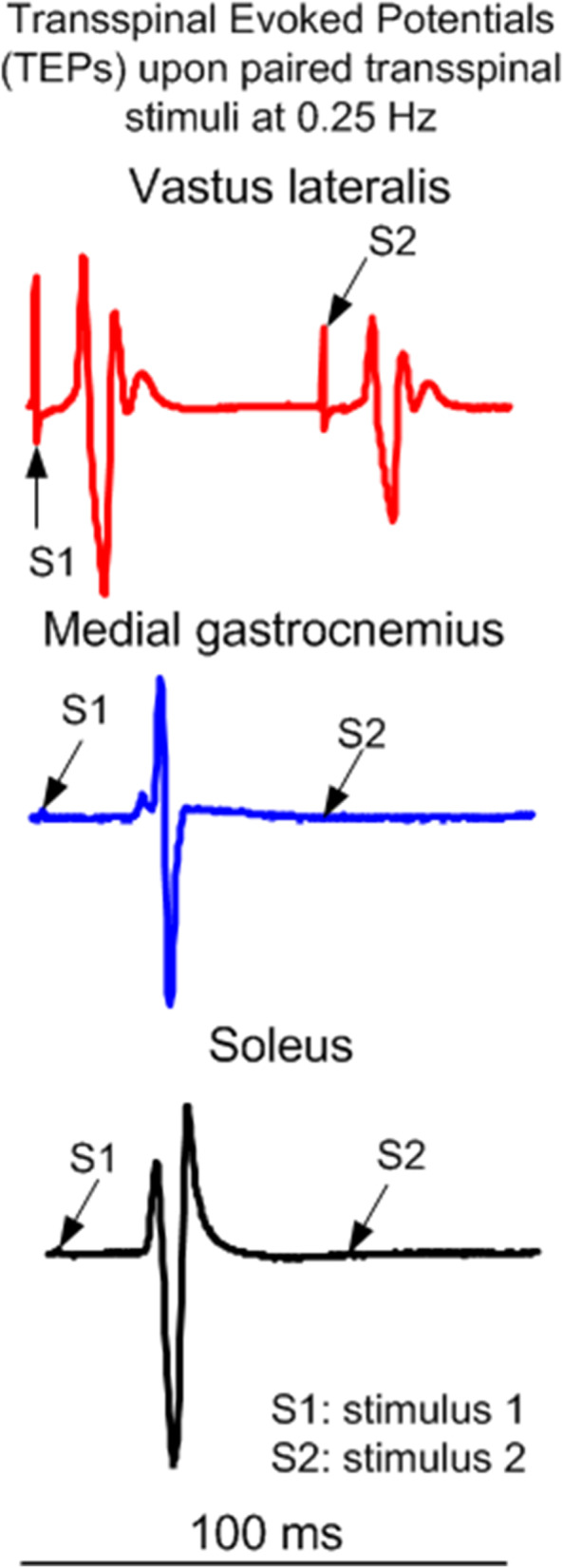
Representative transspinal evoked potentials (TEPs) recorded from the knee (vastus lateralis) and ankle (medial gastrocnemius and soleus) muscles in one person with AIS D SCI lying supine upon paired transspinal stimuli at 60-ms interstimulus interval. Note that the TEP depression is larger in ankle muscles compared to TEPs recorded from the knee muscle

While participants are seated, the Thoracic 10 spinous process is identified via palpation and anatomical landmarks. The upper edge of a single cathode electrode (Uni-Patch™, 10.2 cm × 5.1 cm, Wabasha, MA) is placed at the Thoracic 10 spinous process. Two interconnected electrodes (anode; same type as the cathode), are placed on the abdominal muscles or iliac crests, depending on self-reported levels of comfort. These electrodes are connected to a constant current stimulator (DS7A or DS7AH, DS8R, Digitimer Ltd., UK) that is computer-controlled through analog-to-digital data acquisition boards (National Instruments, Austin TX, USA or 1401 plus running Spike 2, Cambridge Electronics Design Ltd., Hertfordshire, England, UK). Transspinal stimulation is initially delivered as a single monophasic pulse of 1 ms. The intensities corresponding to the soleus TEP threshold and maximal amplitudes are noted on the subject’s data sheet. The soleus TEP amplitude is assessed upon paired transspinal stimuli at 60-ms interstimulus interval to establish susceptibility of TEPs to post-activation depression (Fig. 1). When paired transspinal stimulation does not produce TEP depression, the cathodal electrode position is adjusted. Consistent position of the cathodal transspinal electrode during the intervention is ensured by marking the area with a non-allergenic skin pen.

Once cathodal positioning, thresholds, and TEP depression upon paired stimuli are confirmed, transspinal tonic stimulation is delivered at a frequency of 30 Hz with a DS8R constant current stimulator (charge-balanced, symmetric, biphasic rectangular pulses of a 1-ms width per phase) for 30 min at 1.2 times the soleus TEP threshold. Stimulation intensity is adjusted based on each subject’s reported discomfort. Transspinal stimulation intensity adjustments are made on a weekly basis based on the TEP threshold.

The different body positions for delivery of transspinal stimulation compare efficacy when (1) ongoing neuronal activity is adjusted continuously (standing vs. supine) and (2) upright posture regulation is needed in the presence or absence of transspinal stimulation (active vs. sham during standing). Upright posture regulation, which is greatly affected after SCI, is one of the key elements of locomotor control [55–60]. During standing, local spinal inhibitory circuits continuously adjust the soleus H-reflex amplitude based on body sway amplitude [61]. The soleus H-reflex amplitude is directly related with postural instability and dynamic balance [62] and is susceptible to cortical control [63, 64]. Specifically, upright balance control requires increased soleus MEPs and decreased soleus H-reflexes [65, 66]. In people with SCI, stand training with transspinal stimulation at varying frequencies (0.2 to 30 Hz) promotes self-assisted standing and upright trunk posture with minimal or absent external assistance [67, 68].

**Fig. 2:**
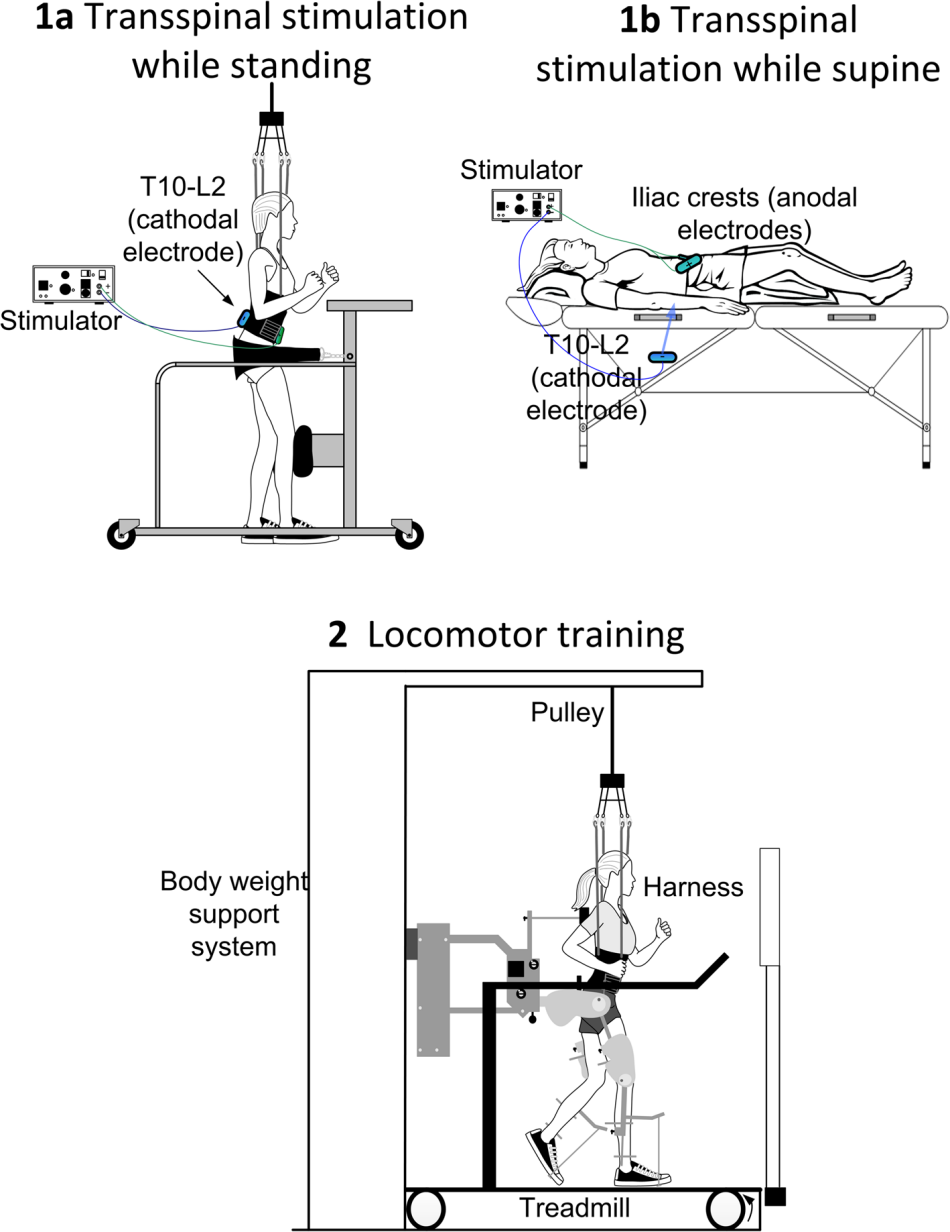
Intervention: Noninvasive transspinal stimulation at 30 Hz for 30 min is delivered during standing (1a) with body weight support as needed to avoid knee buckling and/or while supine with legs semi-flexed at a neutral position (1b) followed by 30 min of locomotor training with the Lokomat 6 Pro (2) within the same training session. In Figs. 1a and 1b the position of the stimulating electrodes is shown

For participants receiving transspinal stimulation while lying supine, hips, and knees are placed in slight flexion and stabilized by holsters and towels to avoid external limb rotations as needed (Fig. 2). For participants receiving active or sham transspinal stimulation while standing, BWS is provided in a standing frame or in the Lokomat to ensure safety (Fig. 2/1a). The initial BWS is adjusted such that knee buckling during standing is absent and decreases over the training course to achieve full body loading. Because prolonged standing may be demanding for individuals with SCI, a 2-min break is given every 10 min of standing.

#### Locomotor training

After 30 min of transspinal stimulation all participants receive 30 min of locomotor training with the Lokomat 6 Pro (Hocoma, Switzerland). Over the training course, we use a clinical algorithm to adjust BWS, ankle straps position, and leg guidance force [26]. The tension of the ankle straps is adjusted based on the right and left TA muscle strength evaluated every week. BWS and leg guidance force are adjusted based on presence or absence of knee buckling during standing. BWS, ankle straps position, and leg guidance force are individualized as is the case during outpatient rehabilitation. All intervention and training sessions are supervised by at least two of the authors.

### Criteria for discontinuing or modifying allocated interventions {11b}

Interventions are discontinued when unanticipated or serious adverse events (UAEs, SAEs) result from the intervention and call into question the safety of the intervention or if any new information that becomes available during the trial necessitates stopping the trial. The criteria for halting a training session or an experiment include subject complaints of shortness of breath, light-headedness, confusion, severe headache, or dyspnea; onset of angina; excessive blood pressure (BP) changes (systolic BP greater than 200 mm Hg, diastolic BP greater than 110 mm Hg, systolic BP less than 80 mm Hg); inappropriate bradycardia (drop in heart rate (HR) greater than 15 beats per min); increased HR exceeding 80% of the predicted maximum HR (HRmax = 220 − age); or participant reports a Borg perceived exertion rate of greater than 15. Should the session be halted, the participant will be asked to rest while BP and HR are monitored and will resume only when BP and HR return to baseline values. If any of these conditions persist after rest, the participant’s primary physician will be contacted, and the participant is referred for evaluation. If the participant complains of angina at rest, loss of consciousness occurs, or cardiac arrest, 911 will be called immediately. There are no special criteria for modifying allocated interventions.

### Strategies to improve adherence to interventions {11c}

To maintain intervention fidelity, we developed and provided to staff members intervention protocols that detail the procedures for conducting the thoracolumbar transspinal stimulation and the locomotor training. To assure the correct implementation of the protocols, we trained staff members to become familiar with the theory and scientific literature supporting the intervention and with the laboratory equipment. We assess the fidelity with which the locomotor training is implemented in terms of compliance with the protocol and adherence to the procedure by recording for every session a participant received, the adjustments of a) BWS, b) leg guidance force, c) treadmill speed and by monitoring continuously participants’ engagement and performance using real-time biofeedback. Additionally, records of the thoracolumbar transspinal stimulation delivered are kept by staff members for each participant. Date, duration, and intensity of stimulation are recorded for every session, while the TEP threshold is established and recorded on a weekly basis. The position of the cathodal stimulation electrode is marked at the first session and checked in every session to ensure that electrode configuration remains the same.

### Relevant concomitant care permitted or prohibited during the trial {11d}

During the trial, enrolled participants are not permitted to participate in another clinical trial and are encouraged to continue their daily routine as before enrollment and participation. We frequently remind participants of the expectations to not initiate other rehabilitation programs during their participation in this study and to keep us informed of any changes in their clinical medication schedules.

### Provisions for post-trial care {30}

Not applicable. All participants benefit from the multiple sessions of locomotor training. There is no increased risk in any of the randomized groups.

### Outcomes {12}

#### Primary outcomes

The primary outcomes are neurophysiological biomarkers that accurately probe corticospinal and spinal neuroplasticity (at presynaptic and postsynaptic motoneuron levels):

1. Corticospinal neuronal excitability:

We establish the active MEP threshold that corresponds to the intensity that evokes 10 responses of at least 50 mV following 20 1-ms transcranial magnetic stimulation (TMS) pulses in seated subjects. Then, the soleus MEP recruitment input–output curve is assembled while maintaining small-amplitude contractions. During BWS-assisted stepping, MEPs are recorded at 1.3 times the right soleus MEP active threshold randomly at different phases of the step cycle (divided into 16 equal bins or time windows) based on the signal from the right foot switch. During stepping, MEPs are collected before and after intervention using Lokomat settings matched to the baseline, while an effort is made to record MEPs using Lokomat settings matched to those of the last training session. This establishes changes at matched settings and adaptations during more demanding stepping conditions. MEPs recorded at different stimulation intensities (recruitment curve) are measured as peak-to-peak amplitude and normalized to the soleus maximal M-wave (Mmax) evoked by posterior tibial nerve stimulation to counteract differences in muscle fiber composition across subjects. Stimulation intensities are normalized to the intensity corresponding to the 50% of the MEPmax amplitude, estimated based on the sigmoid function fit to the data. For the participants who have obtainable soleus MEPs at baseline, the main outcomes from this experiment are the MEPmax from the MEP recruitment curves and the MEP amplitude at the mid-stance and mid-swing phases before and after the intervention.

**Fig. 3:**
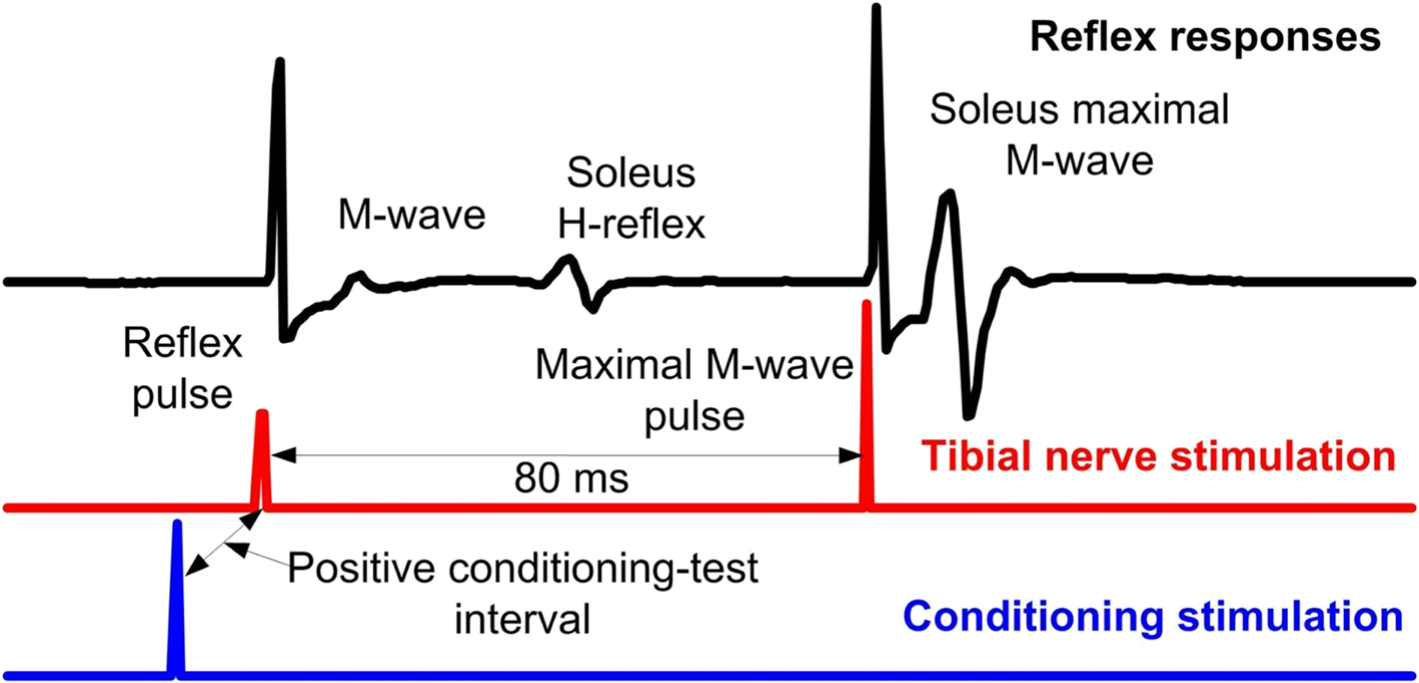
Arrangement of stimuli during stepping. EMG channel, along with reflex and conditioning stimuli are shown as detected in LabVIEW data acquisition software. Test and/or conditioning stimulation (that can be directed to the primary motor cortex, skin, or peripheral nerve as single pulses or pulse trains at variable frequencies) are indicated for an interval that can be adjusted based on the experimental protocol. Through foot switches (not shown), we determine the exact phase that stimulation occurs, i.e., the exact bin. Stimulation is delivered randomly across the 16 bins that make up each step cycle

2) Spinal neuronal excitability:
  *2a) Soleus H-reflex rate-dependent (or homosynaptic) depression*: To assess restoration of homosynaptic depression exerted at the synapses between muscle spindle primary Ia afferents and alpha motoneurons, soleus H-reflexes following posterior tibial nerve stimulation at the popliteal fossa with a 1-ms pulse are recorded from seated subjects at different frequencies (1.0, 0.33, 0.2, 0.125, and 0.1 Hz). Homosynaptic depression is greatest at 1.0 Hz and fully recovers at 0.125 and 0.1 Hz [69]. Homosynaptic depression cannot be recorded during stepping because it is abolished by strong spinal inhibitory circuits and efferent activity. The main outcome measure from this neurophysiological biomarker is the soleus H-reflex 1.0/0.1 Hz ratio [70].
  *2b) Soleus H-reflex depression by antagonistic nerve stimulation*: To examine the restoration of reciprocal inhibitory actions on antagonistic alpha motoneurons, the soleus H-reflex is conditioned by common peroneal nerve (CPN) stimulation at conditioning-test (C-T) intervals of 0, 1, 2, 3 and 4 ms, with subjects seated [71–75]. Reproducible CPN conditioning stimulation is ensured by using a stable, small-amplitude TA M-wave and similar soleus M-wave amplitudes under control and conditioning stimulations. Soleus H-reflexes are measured as peak-to-peak amplitude, accepted for M-waves ranging from 2 to 8% of the maximal M-wave (Mmax), and normalized to the Mmax. The main outcome is the amplitude of the conditioned soleus H-reflex as a percentage of the control H-reflex. The C-T interval for which reciprocal inhibition is present or reciprocal facilitation is the smallest is further investigated during BWS-assisted stepping. The main outcomes are the conditioned H-reflex at rest and during the stance and swing phases of BWS-assisted stepping, which both reflect the amount of reciprocal inhibition.
  *2c) Control and conditioned soleus H-reflexes during stepping*: During standing with BWS as needed, the soleus H-reflex and M-wave recruitment curves are assembled. Then, each participant steps with the assistance of the Lokomat, and soleus H-reflexes are recorded under control conditions and following CPN conditioning stimulation randomly across 16 equal time bins into which each step cycle is divided based on the signal from the foot switch [75]. The tibial nerve stimulation intensity is adjusted in real-time online to evoke H-reflexes such that their corresponding M-waves are 2–8% of the Mmax evoked 80 ms after the test H-reflex at each bin (Fig. 3). Control and conditioned soleus H-reflexes during stepping are collected before (baseline) and 1–2 days after completion of all 40 intervention sessions at Lokomat settings matched to baseline and the last training session. This approach determines changes at matched settings and reflex adaptation at more demanding settings [76]. During stepping, soleus H-reflexes are measured as peak-to-peak amplitude, accepted for M-waves ranging from 2 to 8% of the Mmax, normalized to the Mmax, and averaged for each bin of the step cycle. The main outcome is the control and conditioned soleus H-reflex amplitude normalized to the Mmax at mid-stance and mid-swing phases.

#### Secondary outcomes

The secondary outcomes include the International Standards for Neurological Classification of SCI (ISNCSCI) sensory and motor scores, temperature sensation, proprioceptive sensation, ability to stand and walk, and autonomic function. Standing and balance ability is evaluated with the Berg Balance Scale [77, 78] and BEST tests [79]. Walking capacity is tested with the 2-min and 10-m walking tests [80]. A combination of validated questionnaires related to bladder, bowel, and sexual function are used to assess autonomic function [81–87].

#### Other outcomes

Other outcomes include the recordings of the muscle activation profiles. Locomotor EMG activity is collected to establish changes in the phase-dependent amplitude modulation of muscle activity at bilateral soleus, tibialis anterior, medial gastrocnemius, peroneus longus, medial hamstrings, adductor gracilis, vastus lateralis, and vastus medialis muscles. EMG signals are full wave rectified, band-pass filtered, normalized to the homonymous maximal EMG, plotted against the step cycle, and grouped based on the time of testing and intervention study group. Outcomes are changes in intralimb and interlimb coordination [27].

### Participant timeline {13}

**Fig. 4:**
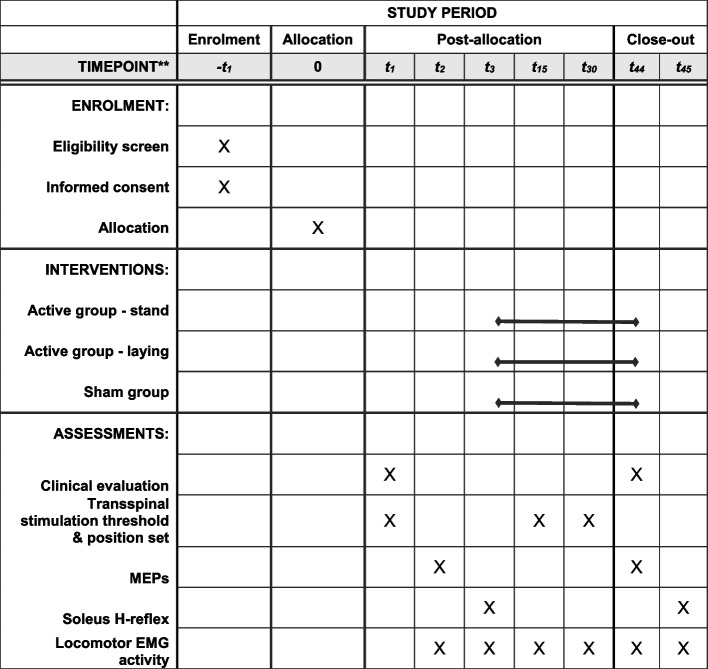
Standard Protocol Items Recommendations for Interventional Trials (SPIRIT) figure indicating the procedures for each participant that occur at each visit. Each participant comes at least 45 times to the lab receiving a total of 40 treatment sessions

The schedule of events for each participant enrolled in this study is presented in Fig. 4. *Enrollment − t*_*1*_*:* Obtain informed consent. Review medical history and medications to determine eligibility based on inclusion/exclusion criteria. Perform medical and neurological examinations needed to determine eligibility based on inclusion/exclusion criteria. Schedule study visits for participants who are eligible and available for the duration of the study. Provide participants with specific instructions needed to prepare for their first study visit, including but not limited to clothing prior to the study. *Baseline visit t*_*1*_*:* Record vital signs. Perform a Lokomat familiarization session. Perform clinical evaluation tests. *Baseline visit t*_*2*_*:* Record vital signs. Record MEPs at rest and during stepping with Lokomat settings obtained from the familiarization session. Provide to the participant the TMS side-effect questionnaire. Record adverse events as reported by participants or observed by investigators. *Baseline visit t*_*3*_*:* Record vital signs. Perform electrophysiological recordings (soleus H-reflex rate-dependent depression and reciprocal inhibition, control, and conditioned soleus H-reflex during BWS-assisted stepping). Record adverse events as reported by participants or observed by investigators. *Intervention visits 4–43:* Administer the assigned transspinal stimulation-locomotor training intervention. Reassess transspinal stimulation threshold and locomotor EMG activity at study Intervention visits *t*_*15*_ and *t*_*30*_. *Final study visits t*_*44*_* and t*_*45*_: Perform clinical evaluation tests and electrophysiological recordings as described in Baseline visits.

### Sample size {14}

Our study is powered to establish and detect a significant effect size across the 3 groups between pre- and post-intervention. We performed statistical analysis of the preliminary data utilizing G*Power-3 software [88, 89]. The sample size was calculated to obtain a power larger than 0.8.

*Aim 1:* Determine if priming locomotor training with transspinal stimulation increases MEP amplitudes and promotes appropriate MEP amplitude modulation during stepping. The TA MEPmax amplitudes recorded in 10 healthy subjects while at rest were compared before and after 10 sessions of transspinal stimulation delivered with subjects lying supine (difference between means: 148.05, pooled standard deviation: 206.8, d = 0.71). To predict sample size at a power of 0.80 at α = 0.05 using repeated measures, within-between interaction ANOVA across 3 groups, 24 total subjects are needed. We will enroll 45 subjects, so we will be adequately powered even if subjects drop out at a higher than expected 20% rate. This sample size is based on results from healthy control subjects and not from SCI subjects.

*Aim 2*: Determine if priming locomotor training with transspinal stimulation restores spinal inhibition, reduces neurophysiological hyperreflexia, and re-establishes H-reflex modulation during stepping. The soleus H-reflex rate-dependent depression in 6 persons with SCI was recorded at 1.0 Hz from the left leg and compared before and after 15 sessions of transspinal stimulation. Based on the soleus H-reflex recorded at 1.0 Hz before and after transspinal stimulation (difference in means of pre versus post: 25.28, pooled standard deviation: 28.92), the effect size was 0.87. To predict sample size at a power of 0.80 at α = 0.05 using a one-way ANOVA across 3 groups, 18 total subjects are needed. We will enroll 45 subjects, so we will be adequately powered even if subjects drop out at a higher than expected 20% rate.

The soleus H-reflex amplitude during assisted stepping in 11 persons with motor incomplete SCI (AIS C-D) recorded from the right leg was compared before and after locomotor training [26]. Based on the amplitude of the soleus H-reflex at mid-stance before and after training (difference in means of pre versus post: 13.67, pooled standard deviation: 17.4), the effect size was 0.79. To predict sample size at a power of 0.80 at α = 0.05 using a one-way ANOVA across 3 groups, 21 total subjects are needed. We will enroll 45 subjects, so we will be adequately powered even if subjects drop out at a higher than expected 20% rate.

Limb coordination relies on the strength of reciprocal inhibition between antagonistic muscles. We used data from a study performed by our group that examined short-latency inhibition from ankle flexor afferents onto ankle extensor motoneurons at rest in 13 persons with AIS C-D SCI before and after locomotor training [28]. Based on the amplitude of the conditioned H-reflex at the conditioning-test interval of 2 ms before and after training (difference in mean of pre versus post: 15.04, pooled standard deviation: 17.21), the effect size was 0.874. To predict sample size at a power of 0.80 at α = 0.05 using a one-way ANOVA across 3 groups, 18 total subjects are needed. We will enroll 45 subjects, so we will be adequately powered even if subjects drop out at a higher than expected 20% rate. Please note that the established power was based on data from SCI subjects who received either transspinal stimulation or locomotor training separately and not combined.

*Aim 3*: Determine if priming locomotor training with high-frequency transspinal stimulation improves limb coordination and the ability to stand and walk. These are secondary measures, and the study is not powered based on these outcomes.

Collectively, an average of 6 to 8 subjects per group is needed to establish significant differences for primary outcomes before and after transspinal stimulation or locomotor training. By enrolling 15 participants per group, the study will be robustly over-powered to detect these differences despite a projected possible 20% dropout rate, sporadic missing data values, or lower-than-expected observed effect sizes.

### Recruitment {15}

In this mechanistic clinical trial, individuals with motor incomplete SCI will participate. In New York and New Jersey, 22,000 people live with SCI, while 600 new cases are reported every year. Given the SCI population within the NY Metropolitan area, recruitment of 45 persons with SCI will not be difficult. We will use diverse and multiple approaches to recruit participants, similar to those we have utilized for our ongoing clinical trials**.** We recruit persons with SCI from (1) the database and outpatients of the James J. Peters Veterans Affairs Medical Center in Bronx, NY, the second performance site, (2) via flyers at the NYC Spinal Cord Injury Association, (3) open houses of SCI Projects at Rutgers, (4) via flyers posted on the lab’s Facebook page, and from (5) outpatient Neurology and Rehabilitation departments of the Mount Sinai Medical Center in New York, NY. In addition, the PIs of the grant reserve booths at the Annual Abilities Expo, New Jersey Convention, and Expo Center every year. We will also use emails from our databases and advertisements in local newspapers to recruit participants.

## Assignment of interventions: allocation

### Sequence generation {16a}

To minimize imbalance between study groups, we performed block randomization with a block size of 9 to assign eligible participants into the 3 groups. If groups are not balanced, we ensure that the following blocked randomized subjects are stratified based on their ability to ambulate with assistive devices or not. We will also check after the completion of intervention by all subjects in each group, whether the groups are balanced or not regarding baseline motor function and perform tests of association accordingly. Blocked randomization is a commonly used randomization technique in clinical trials with a small sample size. This approach reduces bias and fosters balanced allocation of participants into different groups [90].

### Concealment mechanism {16b}

The randomization scheme was generated by a trained biostatistician using R software [91].

### Implementation {16c}

The randomization scheme is accessible only to the biostatistician who generated it (PA). Assignment allocations are released by the biostatistician to other study personnel once each new participant has completed consent and all screening procedures. The sequence of assignments to groups is random and therefore appropriately concealed.

## Assignment of interventions: blinding

### Who will be blinded {17a}

Participants will be blinded to group allocation. Participants allocated to the sham group will receive stimulation at motor threshold intensity for 1 min, followed by a gradual return to no stimulation to mimic the initial sensation of stimulation followed by the acclimation to cutaneous stimulation. The study personnel performing locomotor training, clinical assessments, and data analysis will be blinded to the group allocation. Because each participant’s study code is not linked to the group assignment, those who perform data analysis cannot recognize the study group of a participant.

### Procedure for unblinding if needed {17b}

Staff members who perform the thoracolumbar electrical stimulation and the biostatistician who performs the allocation are not blinded. We do not anticipate any requirement for unblinding but if required, the investigators will have access to group allocations and any unblinding will be reported. Data will be unblinded when data analysis is completed and statistical tests between study groups need to be performed.

## Data collection and management

### Plans for assessment and collection of outcomes {18a}

Neurophysiological biomarkers and clinical outcome assessments at baseline and after 40 sessions of therapy are taken by the same investigator while using the same equipment and settings to ensure consistency and repeatability of recordings and assessments. Laboratory tests/experiments involve recordings of muscle action potentials following brain and/or peripheral nerve stimulation via surface EMG electrodes based on consensus standards for electrophysiological recordings. Consistency of recordings between sessions is ensured from using similar anatomical landmarks, detailed notes on thresholds, amplitude, and behavior of action potentials at a wide variety of stimulation intensities. Data from each participant is checked for completeness through validity checks.

### Plans to promote participant retention and complete follow-up {18b}

Explanation of the study, including possible benefits, increases recruitment and retention in randomized clinical trials [92]. It is a standard procedure in our lab to describe the research study in detail and answer all questions that a participant may have. The eligible participant is always encouraged to ask questions about the procedures and no information is withheld. During the discussion, participants are informed of the expectation that they can commit to traveling to either site for training/intervention sessions at least 4 times per week for up to 3 months. If the participant or study team perceives difficulty in meeting these expectations, then enrollment is deferred. After the consent form is signed, we develop a calendar for each participant based on the time needed to come to the lab, job obligations, or other commitments. The calendar includes all experimental and training sessions for the total duration of the study and is emailed to the participant. We also make use of electronic calendar invitations for reminders, and emails or texts before each visit depending on the participant. These procedures can decrease some common reasons for dropouts in clinical trials such as fear and anxiety during the study, schedule conflicts, forgetting visits, lack of appreciation, and misunderstood expectations. Thus, the procedures that we follow — explaining the importance of participation, setting expectations, reminders of visits, accommodation of the schedule, and promptly responding to inquiries — are expected to contribute to better retention of participants.

### Data management {19}

All participants are given a unique identifying number that is used on all clinical and experimental data for that participant. Clinical data in paper format are kept in a locked file cabinet, while clinical data entered directly from the source documents along with the experimental data are saved with a unique code in password-protected computers.

### Confidentiality {27}

Participant confidentiality is strictly held in trust by all investigators and staff. The study protocol, documentation, data, and all other information generated are held in strict confidence. No information concerning the study or the data are released to any unauthorized third party without prior written approval of the sponsor. Participants are consented individually and participate one at a time, which limits the opportunity for others to become aware of the participant’s participation or responses. All individually identifiable data is protected and only accessible to research staff.

All participants are identified by a study code upon randomization without any identifiable information. The study code is used to save all data in digital forms and written documents, and when data are reported either in a conference or in a research article. Furthermore, signed consent forms and all documents that can identify a participant are kept separate from all forms related to experimental procedures like subject data sheets or post-study questionnaires.

Informed consent documents and medical records: Informed consent is taken in a private, quiet room, and the investigator obtaining informed consent explains procedures thoroughly while asking if the participant has questions at several times during the process. Informed consent documents and medical records are kept separately from all other study data in a locked cabinet at both performance sites. The key is stored in a locked room separate from any of the participants’ data. Only authorized personnel have access to these cabinets.

Email/phone screening information: Information collected during the email or phone screen including names and contact information are entered into a secure database or locked in file cabinets in one of the labs. Once the participant has consented, documents containing protected health information or personally identifiable information are always kept separately from study data linked to the participant’s study code.

### Plans for collection, laboratory evaluation, and storage of biological specimens for genetic or molecular analysis in this trial/future use {33}

None. Not applicable. No biological specimens are collected for research purposes.

## Statistical methods

### Statistical methods for primary and secondary outcomes {20a}

For each dependent variable (primary and/or secondary outcome), descriptive statistics including frequencies and percentages for categorical variables and means and percentile distributions for continuous variables to examine the distribution of outcomes and describe the study population are performed.

We will group each dependent variable based on the time of testing (before and after the intervention) and the subject group (2 experimental, 1 sham). A two-way mixed analysis of variance (ANOVA) along with Bonferroni post hoc* t-*tests [93] at 2 3 levels (2: time, 3: groups) to test the main and interaction effects among subject groups and time will be performed separately for each outcome. Furthermore, repeated measures analysis of covariance (ANCOVA) will be performed for each outcome separately with gender, age, level of SCI, and clinically evaluated baseline walking function as separate covariates. Outcomes will not be analyzed based on SCI severity because the ISNCSCI scale does not correlate with improvements in walking ability [3], while age is considered one of the predictors of ambulation in SCI [94].

Mixed regression methods and analysis of slope and threshold of correlation will be used to establish the relationship between the ability to stand and walk, quality of life, and changes in primary outcomes (neurophysiological biomarkers). We will use an identity or log link based on the data distribution.

### Interim analyses {21b}

No interim analyses are planned.

### Methods for additional analyses (e.g., subgroup analyses) {20b}

There will be no additional analyses.

### Methods in analysis to handle protocol non-adherence and any statistical methods to handle missing data {20c}

Non-adherence to the protocol will be assessed from the intervention log entered by each investigator/clinical coordinator who administers the intervention at each study performance site. If more than 5% of the experimental data values are missing, values will be encoded as − 1 or − 9999, replaced with the mean/median value, encoded as another level of a categorical variable and predictive models that impute the missing data will be applied [95].

### Plans to give access to the full protocol, participant level-data and statistical code {31c}

Deidentified, individual-level data will be deposited to appropriate public repositories such as the Open Data Commons for Spinal Cord Injury (https://scicrunch.org/odc-sci). This will allow meta-analysis of disparate smaller studies; a need which is even more urgent in neurorehabilitation than in other fields that are more amenable to large drug studies.

## Oversight and monitoring

### Composition of the coordinating center and trial steering committee {5d}

The principal investigators (PIs) of this clinical trial are PI # 1, Maria Knikou, PT, MBA, PhD, at the College of Staten Island, City University of New York, and PI # 2, Noam Y. Harel, MD, PhD, at Icahn School of Medicine at Mount Sinai and James J. Peters Veterans Affairs Medical Research Center (JJPVAMC). PI # 1 performs along with her research team at the Klab4Recovery all neurophysiological recordings before and after training for all study participants of the clinical trial to ensure consistent experimental procedures for all neurophysiological recordings. PI # 1 also leads the implementation of transspinal stimulation and locomotor training interventions, as well as the clinical assessments before and after training for study participants at Klab4Recovery. PI # 2 leads the implementation of transspinal stimulation and locomotor training interventions as well as the clinical assessments before and after training for study participants at JJPVAMC. Both PIs ensure that locomotor training and transspinal stimulation are implemented equivalently at both sites. The trial steering committee is composed of the PIs and dedicated research staff.

### Composition of the data monitoring committee, its role and reporting structure {21a}

The data and safety monitoring board (DSMB) committee includes 4 faculty from the City University of New York and are IRB-approved to serve as members of the DSMB. The DSMB members provide description of stopping rules and justification for the stopping rules, prospectively identified criteria for discontinuing the study, and determination of UEs, AEs, and SAEs. The DSMB committee is independent from the sponsor.

### Adverse event reporting and harms {22}

Recording of AEs, SAEs, serious unexpected adverse events (SUAEs), or unanticipated problems (UPs) occurs at all visits as reported by a participant or observed by study personnel and reported to the PIs. All events are recorded, while SAEs, SUAEs, and UPs are reported immediately to the IRB and DSMB. AEs are recorded and submitted to the study sponsor and to the reviewing IRB at the next annual review date. In case of occurrence or report of a SUAE, the PIs will complete an SUAE form and submit to the study sponsor and to the reviewing IRB as soon as possible, but within 5 working days after the PI first learns of the incident.

For incidents or events that meet the Office for Human Research Protections (OHRP) criteria for UPs, a UP report form will be completed and submitted to the IRB and to the NIH. The UP report will include the following information: protocol identifying information: protocol title and number, PIs name, and the IRB project number; a detailed description of the event, incident, experience, or outcome; an explanation of the basis for determining that the event, incident, experience, or outcome represents an UP and whether it is probably, possibly, or unlikely to be study-related; a description of any changes to the protocol or other corrective actions that have been taken or are proposed in response to the UP. To satisfy the requirement for prompt reporting, UPs will be reported using the following timeline: UPs that are SAEs will be reported to the IRB and to the NIH within two days of the investigator becoming aware of the event. Any other UP will be reported to the IRB and to the study sponsor within ten days of the investigator becoming aware of the problem. All UPs will be reported to appropriate institutional officials (as required by an institution’s written reporting procedures), the supporting agency head (or designee), and OHRP within 10 days of the IR’s receipt of the report of the problem from the investigator. Both PIs will be responsible for ensuring participants’ safety on a daily basis and for reporting all adverse events AE and UP to the IRB.

Based on our extensive clinical and research experience with SCI, transspinal stimulation, and locomotor training, we do not anticipate any SUAEs to occur. No safety issues have been reported following transcutaneous tibial nerve stimulation or following transcutaneous spinal cord (or transspinal) stimulation. In 38 out of 89 persons with SCI who received locomotor training with the Lokomat, adverse events included mild skin erythema at the sites of the cuffs, and muscle pain while open skin lesions (*n* = 2), joint pain (*n* = 2), or tendinopathy (*n* = 1) were also reported [96]. The events are predictable and are indicated as potential risks in the Informed Consent Form.

### Frequency and plans for auditing trial conduct {23}

Both PIs are responsible for ensuring participants’ safety daily and act in an advisory capacity to the NIH to monitor participant safety, evaluate the progress of the study, to review procedures for maintaining the confidentiality of data, the quality of data collection, management, and analyses. The PIs meet annually with the DSMB committee to review trial conduct.

Both PIs ensure that the experimental and training protocols are conducted as pre-determined and are responsible for the integrity and quality control of data. Quality control includes regular data verification and protocol compliance checks. All clinical data (including AEs, concomitant medications, and expected adverse reactions data) and clinical-laboratory data are entered into a 21 CFR Part 11-compliant data capture system. The data capture system includes internal quality checks, such as automatic range checks, to identify data that appear inconsistent, incomplete, or inaccurate. Monitoring of data is performed at both performance sites to ensure that the rights and well-being of trial participants are protected, that the reported trial data are accurate, complete, and verifiable, and that the conduct of the trial is in compliance with the approved protocol/amendment(s). Each site performs internal quality management of study conduct, data collection, documentation, and completion. An individualized quality management plan is developed to describe each site’s quality management. Internal audit is conducted by research staff. Meetings between the PI and research staff at each site hold at regular intervals and not less than once a week.

### Plans for communicating important protocol amendments to relevant parties (e.g., trial participants, ethical committees) {25}

Protocol amendments will be submitted for approval first to the Program Officer of the NICHD/NIH and then to the Institutional Review Board (IRB) before implementation. Following IRB approval, protocol amendments will also be submitted to ClinicalTrials.gov. Changes will be communicated to all investigators.

### Dissemination plans {31a}

#### Research findings and products

Research findings will be disseminated as publications in high-impact peer-reviewed academic journals and research summaries for clinical professional journals to provide the following milestones in clinical translational research. These milestones involve the identification of benefits (and drawbacks) by priming locomotor training with noninvasive transspinal stimulation in individuals with chronic SCI, and on the effectiveness of combined interventions on recovery of standing and walking ability in individuals with chronic SCI. Furthermore, a better understanding of the impact of transspinal stimulation based on body position will be provided. In addition to peer-reviewed publications, the results of the clinical trial will be submitted to ClinicalTrials.gov in compliance with the NIH policy requirements.

#### Dissemination goals

We aim to disseminate the findings of this study to scientists and patients and frontline clinical providers with the goal of improving quality of care, because noninvasive transspinal stimulation can be quickly transferred into different real-world clinical settings to reduce motor dysfunction after SCI and other upper motoneuron lesions in humans. Further, transspinal stimulation will be administered to patients in conjunction with locomotor training, supporting concomitant utilization of multiple interventions as occurs in real-life clinical rehabilitation settings.

#### Target audiences

There are four key audiences for this research: (1) patients and the public; (2) academia; (3) family physicians, physiatrists, physical therapists, and other frontline clinical staff; (4) external statutory organizations such as the Department of Health and the NIH. The PI has already developed and utilized a plan of targeting these end users by providing in-service research talks to local hospitals (4/year), hosting a research booth at the Abilities Expo in New Jersey attended by physicians, therapists, care givers, and patients. A barrier in trying to implement transspinal stimulation as treatment is that therapists and clinicians will require some basic form of training, which can be overcome by workshops.

#### Key message and communication avenues

The key message of this research study is the use of noninvasive transspinal stimulation to increase the effectiveness of traditional rehabilitation such as locomotor training in individuals with SCI and probably for other types of upper motoneuron lesions. This will change the standard of care promoting noninvasive approaches of rehabilitation relying on scientific evidence and not on anecdotal observations. We will communicate our key findings via the following avenues: *Broadcast media*: academic journals, book chapters, technical reports, regular newspapers, special interest newsletters, radio, or television interviews, websites, and social media such as Twitter, local and national SCI foundation newsletters, development of links with key SCI organizations. *Personal contact*: clinical specialty associations, informal professional networks, professional conferences, professional meetings (e.g., grand rounds), workshops, and other continuing medical education training.

#### Evaluation

We will evaluate the success of our team’s dissemination efforts based on the number of website hits, number of inquiries received, and number of physicians/clinicians responses to advertisements. We will obtain feedback on what is needed to translate research findings into practice in their setting.

#### Dissemination strategy summary

The research product of this study is the use of noninvasive transspinal stimulation along with locomotor training for individuals with spinal cord injuries or other upper motoneuron lesions. It can be used to maximize the benefits of traditional rehabilitation, and due to its noninvasive approach, it can be implemented in different real-life clinical settings worldwide. Our primary end users are patients, family physicians, physical therapists, hospital administrators, and medical schools. We plan to involve users in our dissemination efforts by leaflets written in lay language, demonstration of the intervention, and workshops. We will use the Christopher and Dana Reeve Research Foundation, Spinal Cord Injury Research Board of the New York State Department of Health, United Spinal Association—New York Chapter in Queens, New York, to help us disseminate our research product. We will communicate the results via publications in peer-reviewed journals, broadcast media avenues, and personal contact as outlined above. Potential obstacles that we face in disseminating the research findings and product include the skills required to implement our intervention in clinical settings. We will mitigate these obstacles via demonstration and clinical workshops organized at both research sites. We plan to evaluate the dissemination plan based on the number of inquiries received from the public, physicians, and patients, and we will encourage feedback from patients and clinicians. Responsibilities of the above-described dissemination plan will be equally distributed among all research team members. Specifically, both PI’s will be responsible for dissemination to hospitals and local-national SCI associations/foundations and publication of results in ClinicalTrials.gov, while the development of leaflets, website maintenance, and Abilities Expo will each be organized by 2 research associates.

## Discussion

Physical rehabilitation after SCI offers benefits, but rehabilitation alone is not enough to fully engage the plasticity needed to repair the nervous system. In this clinical trial, we apply non-invasive transspinal electrical stimulation to prime the nervous system before each session of physical rehabilitation (locomotor training). We use comprehensive neurophysiological and clinical assessments to measure recovery of nerve transmission, muscle reflex coordination, standing, walking, and quality of life. Understanding how transspinal stimulation augments exercise-based plasticity from neurophysiological and clinical perspectives is extremely important for the development of targeted and tailored neuromodulation interventions for SCI. Thus, this clinical trial will greatly impact clinical practice. This is because in real-world clinical settings, noninvasive transspinal stimulation can be more easily and widely implemented than invasive epidural stimulation. Additionally, by applying multiple interventions to accelerate motor recovery, we are employing a treatment regimen that resembles a traditional clinical approach, while administering two noninvasive therapeutic modalities that may have the ability to synergistically restore standing, walking, and other essential clinical functions in SCI.

## Trial status

This is the first protocol of the clinical trial with Identifier NCT04807764. Recruitment started in March 2021 and will be completed in approximately April 2025. Protocol amendment number: 2.

## Supplementary Information

**Additional file 1.**

## Data Availability

Both PIs have access to all trial datasets in digital and/or paper formats. Investigators who work in the project as clinical coordinators, fellows, and research volunteers have access to the data pertaining to data analysis for different avenues of publications, but their access to data ends when their appointment is ended. Data sharing is not applicable to this article as no datasets were generated or analyzed during the current study. Any material required to support the protocol can be supplied on request.

## Abbreviations

AE: Adverse event
ANOVA: Analysis of variance
ANCOVA: Analysis of covariance
AIS: American Spinal Injury Association Impairment Scale
BP: Blood pressure
BWS: Body weight support
CPN: Common peroneal nerve
C-T: Conditioning-test
DSMB: Data and safety monitoring board
EMG: Electromyography
HR: Heart rate
H-reflex: Hoffmann reflex
IRB: Institutional Review Board
ISNCSCI: International Standards for Neurological Classification of Spinal Cord Injury
JJPVAMC: James J. Peters Veterans Affairs Medical Research Center
MEP: Motor evoked potential
Mmax: Maximal M-wave
NCMRR: National Center for Medical Rehabilitation Research
NICHD: National Institute of Child Health and Human Development
NIH: National Institutes of Health
NSCISC: National Spinal Cord Injury Statistical Center
OHRP: Office for Human Research Protections
PI: Principal investigator
SAE: Serious adverse event
SCI: Spinal cord injury
SUAE: Serious unexpected adverse event
TA: Tibialis anterior
TEP: Transspinal evoked potential
TMS: Transcortical magnetic stimulation
UAE: Unanticipated adverse event
UP: Unanticipated problem
