# Supplementary material for: Priming locomotor training with transspinal stimulation in people with spinal cord injury: study protocol of a randomized clinical trial
Source: Trials. 2023 Feb 25;24:145. doi: 10.1186/s13063-023-07193-4 (PMC9960224; doi:10.1186/s13063-023-07193-4)
Supplement: Supplementary file 1 — Additional file 1. [file 13063_2023_7193_MOESM1_ESM.docx]

THE CITY UNIVERSITY OF NEW YORK

*College of Staten Island*

*Department of Physical Therapy/School of Health Sciences*

# CONSENT TO PARTICIPATE IN A RESEARCH STUDY

**Title of Research Study:** Priming with high-frequency transspinal stimulation to augment locomotor training benefits in spinal cord injury

**Principal Investigator:** Maria Knikou, PT, PhD

Professor

**Research Sponsor**: National Institute of Child and Human Development (NICHD)

National Institutes of Health (NIH)

You are being asked to participate in a research study because you are an adult with a diagnosis of spinal cord injury (SCI), and there are no medical or health reasons that do not allow you to receive stimulation over your back and robotic gait training.

This is a randomized multi-site clinical trial performed at two different sites. The first site is at the Klab4Recovery, a research lab directed by Dr. Maria Knikou located at the College of Staten Island campus. At the 2^nd^ performance site, Dr. Noam Harel directs the clinical trial at the Spinal Cord Damage Center at the James J. Peters VA Medical Center in the Bronx. If you are randomized to participate at the CSI-site you will not be visiting the VA-site. If you are randomized to participate at the VA-site you will receive the training intervention sessions at the VA-site but you will come to the CSI-site to participate in a single session (testing visit) during which we will take neurophysiological measures before and after the training intervention to establish recovery of motor function. If you come to both sites you will sign the consent forms from both sites.

**Purpose:**

# The purpose of this research study is to establish recovery of movement and sensation of the legs following stimulation over the back and robotic gait training in people with SCI. Robotic gait training means that you will be walking on a moving treadmill with body weight support while the movement of your legs will be assisted by motor-driven leg braces. Stimulation over the back will be delivered while you are standing with the help of an upper body harness or while you lay on your back. It is also possible that you may enroll in the group that will receive robotic gait training with not active stimulation over the back. Stimulation will be delivered for 30 minutes that will be followed by 30 minutes of robotic gait training.

**Key Information:**

- The consent is being sought for research purposes only, and participation is voluntary.
- The purpose of the research is to provide clinical and scientific evidence on the potential benefits of stimulation over the low back when combined with robotic gait training in individuals with SCI.
- The expected duration of each prospective subject's participation is 9 consecutive weeks and involves 44 consecutive visits, excluding weekends and holidays.
- The foreseeable risks or discomforts include discomfort by the stimulation over the low back or nerve behind the knee, sensation of pressure on the abdominal area by the upper body harness, and sensation of pressure in the legs by the motor-driven leg braces.
- There might be no direct benefit to you or to others. However, stimulation and gait training have been shown to improve standing and walking performance as well as being beneficial for maintenance of bone and muscle integrity after SCI.
- This research study is NOT a treatment.
- The alternative is not to participate in the research study.

**Procedures:**

If you volunteer to participate in this research study, we will ask you to do the following: You will come to the Klab4Recovery research lab located at the 2800 Victory Blvd, Building 5N, Room 218, College of Staten Island. On the first and last two days of your participation, you will stay in the lab for 3 hours, while the other days you will stay for 1 hour. You will come at times previously arranged for you based on your schedule.

In this study, we will use a constant current stimulator (DS7A, Digitimer, United Kingdom) to stimulate your back during your daily sessions of stimulation followed by robotic gait training. We will use the same stimulator to stimulate a nerve in the back of your knee at the beginning and at the end of the study to describe changes in the nervous system. We will also use a transcranial magnetic stimulator (MagStim, MA, USA) to stimulate your brain. The US Food and Drug Administration (FDA) has approved the MagStim device to treat for example obsessive compulsive disorder, major depression, or migraine headaches delivered repeatedly at specific frequencies or for stimulation of peripheral nerves. We will use the MagStim device for investigational purposes by sending single pulses to the brain to evoke responses that will be recorded with surface pads from your leg muscles. These responses will detect changes in your nervous system after the intervention.

**PRE-TRAINING TESTING VISITS 1 and 2 (3 hours each):**

At the first and last two days you will need to bring or wear shorts that are loose in the legs in order for us to place pads on your legs to record the twitches of your muscles following stimulation.

Initially, we will measure your blood pressure while you are seated and will assess your sensation, movement of your legs and ability to walk via standard clinical tests. This will be repeated also after you complete all training sessions.

Then, the skin on the front and back of your legs will be dry shaved (if needed), and cleaned with alcohol pads. Next, we will place pads (known as surface electrodes) on the front and back parts of your legs. These pads will be secured with non-allergenic sterilized tape. These pads will record the twitches from your leg muscles when the nerve in the back of your knee, ankle, and the skin of your back at your waist level are stimulated. These pads will also record the twitches of your leg muscles when transcranial magnetic stimulation is delivered to your head to stimulate your brain.

Then, a pair of pads will be placed on the back and in front of your waist. These pads will deliver stimulation to the spinal cord, and you will feel twitches in your leg muscles. None of the stimulation is painful, but if you feel discomfort we will decrease the intensity or stop altogether. At that point, you can decide to take a break and continue when you are ready or you can end participation in the study if you wish. The nerves or the skin over your back will be stimulated with a single pulse and 20 stimulations will be delivered. This will be repeated no more than 10 times.

Then, while you are seated, a magnetic coil connected to a device called transcranial magnetic stimulation will be placed over your head (Seated Magnetic testing). When the magnetic coil is placed at specific sites over the head, it stimulates the brain and produces twitches in arm or leg muscles. You will wear ear plugs and a mouth guard to feel comfortable during this procedure. While seated, we will move the magnetic coil at different sites on your head. Stimulation will be delivered once every 10 seconds. When this is completed, we will stimulate at different intensities and we will record about 100 twitches from your leg muscles. We will not record twitches from your arm muscles.

The second day, we will place again pads on the skin of your legs and you will transfer to the treadmill of the robotic device known as Lokomat®. Full body weight support will be provided to you based on your ability to stand. Similarly, the leg braces will assist you to step based on your ability to move your legs. Then, you will start walking with body weight support and your legs movement will be assisted by the motor-driven leg braces. You will walk for a few minutes to get used to the treadmill. When you will be walking on the treadmill, we will deliver low-intensity stimulation to the nerve behind the knee in your right leg every 5 steps. We will also deliver low-intensity stimulation to your back when you will be walking on the treadmill, and at the skin of your lower leg. Stimulations will produce twitches in your leg muscles that you will not be able to stop them.

Stimulation may be uncomfortable and may produce some discomfort but the intensity will be low in amplitude, similar to that utilized during standard clinical electrophysiological tests used for diagnosis. Treadmill speed will be set at a value that you feel most comfortable to step.

**SPINAL STIMULATION AND ROBOTIC GAIT TRAINING:**

**VISITS 3 THROUGH 42 (1 hour each, 40 total sessions of training):**

During these visits, and based on the group you have been enrolled, you will receive non-invasive spinal stimulation for 30 minutes while standing or while lying supine, or you will receive sham stimulation for 30 minutes during standing. Transspinal tonic stimulation will be delivered at a frequency of 30 Hz (charge-balanced, symmetric, biphasic rectangular pulses of a 1-ms width per phase) at 3 times the soleus muscle motor threshold. Stimulation may be uncomfortable but the intensity will be lowered, if necessary, based on your comfort level.

When the stimulation time is completed, you will receive for 30 minutes assisted treadmill walking on the robotic device (Lokomat®). The robotic device will provide, as needed, leg guidance and body weight support via an upper body harness. The robotic-assisted treadmill walking will be similar to the procedure described above in the Testing Visits. Each training session will last 1 hour. Treadmill speed, body weight support and leg guidance force by the robotic device will be set when you feel most comfortable to step.

**POST-TRAINING TESTING: VISITS 43-44 (3 hours each)**

Using similar techniques as described above in the Testing Visits 1-2, we will record your muscle twitches in response to stimulation of your nerves and brain while seated, and the muscles twitches of your legs during walking with the robotic device. We will also repeat the clinical assessment of your leg muscle tone, leg muscle strength, and your walking capacity (if applicable) during the testing visit.

**Audio Recording/Video Recording/Photographs:**

We will video record you during robotic gait training, in order to document the position of the trunk and the movements of your legs during your training sessions. The position of the trunk and the movements of your legs will be reviewed by the research team. You can still participate in this study if you do not consent to video recording.

**Time Commitment:**

Your participation in this research study is expected to last for a total of 9 consecutive weeks. You will not have to come during weekends and holidays*.*

**Potential Risks or Discomforts:**

There is a small risk of feeling uncomfortable with stimulation delivered either to your low back or to the nerve behind your knee.

- - The study team members are trained and experienced in stimulation of nerves found in the legs, and recording responses from your leg muscles upon stimulation. If you feel discomfort, we will decrease the stimulation intensity and will give you breaks.

There is a small risk of falling during transfers between wheelchairs and treadmills; with the risk of falling is the risk of bone fracture.

- - The study team members are trained and experienced in the care and rehabilitation of participants with limited mobility, including extensive safety experience in preventing falls. You will never be left unattended during a test or training session.

There is a possible risk that electrical stimulation or treadmill walking may cause an “autonomic response” – this could include symptoms such as nausea, light-headedness, and sweating. It may even cause you to faint. If this happens, we will immediately stop the procedure. The symptoms should resolve within seconds of stopping electrical stimulation.

- - The investigators will be monitoring your blood pressure, heart rate, and symptoms during all visits. If there are significant symptoms with changes in vital signs, they will halt the procedure immediately.

There is a possible risk for blood pressure variation with stimulation or orthostatic hypotension because some participants are immobile.

- - To counteract this potential risk, we will monitor your blood pressure at every training and testing session.

There is a small risk of seizure from the magnetic stimulation (Testing visits only).

- - This risk is small, and has only been reported for participants undergoing repetitive magnetic stimulation – you will only be receiving one magnetic pulse at a time, so the risk is even smaller.
  - Before you begin participation in the Study, we will screen you for seizure risk, such as history of prior brain injury, or taking certain medications that raise the risk of seizure. If your seizure risk is too high, you will not be able to participate in the Study.
  - The investigator conducting the magnetic stimulation, Dr. Maria Knikou, is a neurophysiologist with extensive experience in this type of stimulation.

There is a risk of headache or ear tingling from the magnetic stimulation (Testing visits only).

- - The possibility of mild temporary headaches is sometimes reported after magnetic stimulation. This risk will be minimized by taking breaks during the stimulation part of the protocol.
  - The magnet makes a clicking noise that can irritate the ears. This will be minimized by using earplugs during magnetic stimulation.

**Potential Benefits:**

There are no direct benefits to you by participating in this study. However, findings from this research study may assist to develop better rehabilitation strategies in the future for people with SCI.

**Alternatives to Participation:**

The alternative is not to participate. All participants with SCI in this study will get physical exercise by robotic gait training. During study participation, you will be asked to not undergo any *new* physical treatments or other clinical research outside of this study. No routine clinical care or medications that you are scheduled for will be withheld from you. You have the option not to participate in this study.

**Participation in other rehabilitation treatments or research studies**:

During the duration of the clinical trial study, you will continue your daily routine as usual. You will continue stretching or standing, if that’s what your daily routine involves. You will not participate concomitantly in another research clinical trial. Also, you should not receive regular rehab treatment for the duration of the study. Since it is more than 6 months from the time you had the spinal cord injury, participation in this study will not affect your current recovery status. Stimulation of skin, nerves, and brain are used in many patients, but we do not know in advance how the stimulation will affect your recovery.

**Payment for Participation:**

You will receive $100 for each testing and clinical evaluation session (3 sessions on 3 different days at the beginning and at the end of the study: 6 sessions x $100=$600), and $35 for each training session (40 sessions x $35=$1400). The total amount of stipend will not exceed $2,000 if you complete all testing and training sessions. You will receive stipends in cash, based on your preference, at the end of each visit, at the end of each week, or at the end of the study. You will receive the stipend in a closed envelope.

**Research Related Injury**

If you become ill or injured as a result of the study (devices or procedures), seek medical treatment through your doctor or treatment center of choice. You will not be reimbursed for these expenses. Promptly tell Dr. Knikou about any illness or injury.

**New Information:**

You will be notified about any new information regarding this study that may affect your willingness to participate in a timely manner.

**Confidentiality:**

We will make our best efforts to maintain confidentiality of any information that is collected during this research study, and that can identify you. We will disclose this information only with your permission or as required by law.

We will protect your confidentiality by giving you a subject code. We will not use your name or other identifying information. The recordings and the results of your tests will be stored by a subject code in a personal computer at Dr. Maria Knikou’s office located on the second floor, of building 5-North, Room 218 at the College of Staten Island that is password protected. Any paper records including health information and medical records will be kept in a locked file cabinet at the Principal Investigator's office. Only researchers involved with this study will have access to this information.

Results of this study may be used for teaching, research, publications, or presentations at scientific meetings. If your individual results are discussed, your identity will be protected and not disclosed. The only people who will know that you are a research subject are members of the research team and, if appropriate your physicians and nurses. No information about you, or provided by you during the research, will be disclosed to others without your written permission, except if necessary to protect your rights or welfare (for example, if you are injured and need emergency care), or if required by law.

The research team, authorized CUNY staff, the research sponsor (NICHD/NIH) that funds this clinical research trial, and members of the Data Safety and Monitoring Board panel may have access to research data and records in order to monitor the research. Research records provided to authorized CUNY and non-CUNY individuals will not contain identifiable information about you. Publications and/or presentations that result from this study will not identify you by name.

The information we collect from you as part of this study will not be used or distributed for future research.

**Participants’ Rights:**

- Your participation in this research study is entirely **voluntary**. If you decide not to participate, there will be no penalty to you, and you will not lose any benefits to which you are otherwise entitled.
- You can decide to withdraw your consent and stop participating in the research at any time, without any penalty.

**Questions, Comments or Concerns:**

If you have any questions, comments or concerns about the research, you can talk to one of the following researchers:

- Dr. Maria Knikou at 718-982-3316, Monday through Friday, from 9 am to 5 pm and at 718-687-2893 after office hours.
- Mr. Shammah Solomon or Dr. Andreas Skiadopoulos at 718-982-2821, Monday through Friday, from 9 am to 5 pm.

If you have questions about your rights as a research participant, or you have comments or concerns that you would like to discuss with someone other than the researchers, please call the CUNY Research Compliance Administrator at 646-664-8918 or email [HRPP@cuny.edu](mailto:HRPP@cuny.edu).

Alternately, you can write to:

CUNY Office of the Vice Chancellor for Research

Attn: Research Compliance Administrator

205 East 42^nd^ Street

New York, NY 10017

**Participant Signature for Audio/Video Recording:**

If you agree to audio recording/video recording*,* please indicate this below.

_________ I agree to audio recording/video recording*.*

**_________** I do **NOT** agree to audio recording/ video recording.

**Signature of Participant:**

If you agree to participate in this research study, please sign and date below. You will be given a copy of this consent form to keep.

_____________________________________________________

Printed Name of Participant

_____________________________________________________ __________________________

Signature of Participant Date

**Signature of Individual Obtaining Consent:**

_____________________________________________________

Printed Name of Individual Obtaining Consent

_____________________________________________________ __________________________

Signature of Individual Obtaining Consent Date
